# Parkinson's disease-associated genetic variation is linked to quantitative expression of inflammatory genes

**DOI:** 10.1371/journal.pone.0175882

**Published:** 2017-04-13

**Authors:** Steven Pierce, Gerhard A. Coetzee

**Affiliations:** Center for Neurodegenerative Science, Van Andel Research Institute, Grand Rapids, MI, United States; Huazhong Normal University, CHINA

## Abstract

Genome-wide association studies (GWAS) have linked dozens of single nucleotide polymorphisms (SNPs) with Parkinson’s disease (PD) risk. Ascertaining the functional and eventual causal mechanisms underlying these relationships has proven difficult. The majority of risk SNPs, and nearby SNPs in linkage disequilibrium (LD), are found in intergenic or intronic regions and confer risk through allele-dependent expression of multiple unknown target genes. Combining GWAS results with publicly available GTEx data, generated through eQTL (expression quantitative trait loci) identification studies, enables a direct association of SNPs to gene expression levels and aids in narrowing the large population of potential genetic targets for hypothesis-driven experimental cell biology. Separately, overlapping of SNPs with putative enhancer segmentations can strengthen target filtering. We report here the results of analyzing 7,607 PD risk SNPs along with an additional 23,759 high linkage disequilibrium-associated variants paired with eQTL gene expression. We found that enrichment analysis on the set of genes following target filtering pointed to a single large LD block at *6p21* that contained multiple *HLA*-MHC-II genes. These MHC-II genes remain associated with PD when the genes were filtered for correlation between GWAS significance and eQTL levels, strongly indicating a direct effect on PD etiology.

## Introduction

Parkinson’s disease [MIM 168600] is an age-associated, incurable neurodegenerative disorder with a cumulative lifetime risk of close to 10% [1]. Most cases are genetically complex and sporadic, though twin studies indicate heritability is around 34–60% [2]. In addition to 7 autosomal and recessive monogenic causal genes, at least 24 loci have been associated by GWAS with an increased risk of developing Parkinson’s [3, 4]. Depending on the method of association, these loci link Parkinson’s disease etiology with a set of between 24 and 800 genes having specific activity in a multitude of tissues.

Recently, some progress has been made in experimentally linking specific risk polymorphisms with allele-specific regulatory activity and corresponding neighboring gene activity [5]. However, even in this best-studied case, the exact impact of altered expression to the neighboring gene, *SNCA* [MIM 163890], on PD remains unclear. For the majority of risk loci, the specific disrupted-regulatory element and related gene expression changes are unknown, preventing experimental manipulation of the sort seen in [5].

Previously, we examined the nonrandom distribution of PD risk SNPs overlapping tissue-specific putative regulatory elements (REs) [6]. That analysis indicated the tissue or tissues in which an allele-specific effect was most likely to be causal. Surprisingly, most RE enrichment was not seen in brain tissue, indicating that these loci may confer a predisposition to PD through biological effects that are remote from the eventual symptomatic tissues. One draw-back of this method, however, is that it did not link risk to specific gene sets but only to tissue-specific active regions generally. Furthermore, the specifics of the enrichment excluded several major loci from analysis. In the present study, we examine different sets of genes potentially associated with PD risk loci in order to identify common functional pathways. In order to find experimental genetic targets, we linked genes to nearby super-enhancers by SNPs in a comprehensive genome-wide screen.

## Materials and methods

### PD risk variants

Genetic polymorphisms, which have been associated directly or by imputation with PD, were obtained from pdgene.org (p. value <0.0001), PheGenI, and GWAS catalog on 7/2016 providing 7,607 significant risk index sites. Finding further proxy variants in linkage disequilibrium (LD), r^2^ > 0.8 in using rAggr added an additional 23,759 variants (the majority of which were single nucleotide polymorphisms (SNPs). For convenience, we referred to all as risk SNPs. Offspring LD-SNPs were risk-annotated according to the most significant parent GWAS p-value when multiple linked SNPs were present.

### Analysis

Significant eQTLs were downloaded from GTEx July 2016, version 6. Intersection with risk SNPs was determined using custom Perl scripts to generate matched gene/SNP eQTLs according to rsID. Tissue source for eQTL values were annotated. Cleaned txt files were then analyzed using the statistical package, R. Correlation between GWAS significance and eQTL expression was calculated and subset accordingly. Custom Perl scripts were used to find overlap between dbSUPER defined enhancer regions and risk SNPs. Gene set enrichment analysis on egenes associated with risk SNPs was performed using a variety of GSEA software, including DAVID, Metacore from Thomson Reuters, Gorilla, PANTHER, which all gave broadly similar results. Query sets were compared against a background of all significant egenes in GTEx when possible during GSEA. TF binding motif-disruption was found via the R package MotifBreakR, for Factorbook motifs, searched against the dbSNP build 142 and dbSNP build 144. Directional associations for pdgene.org risk SNPs and eQTLs at the HLA locus were oriented according to the GTEx measured minor allele frequency, correlated according to gene by GWAS OR vs. eQTL beta value, and averaged across tissues.

## Results

**Fig 1:**
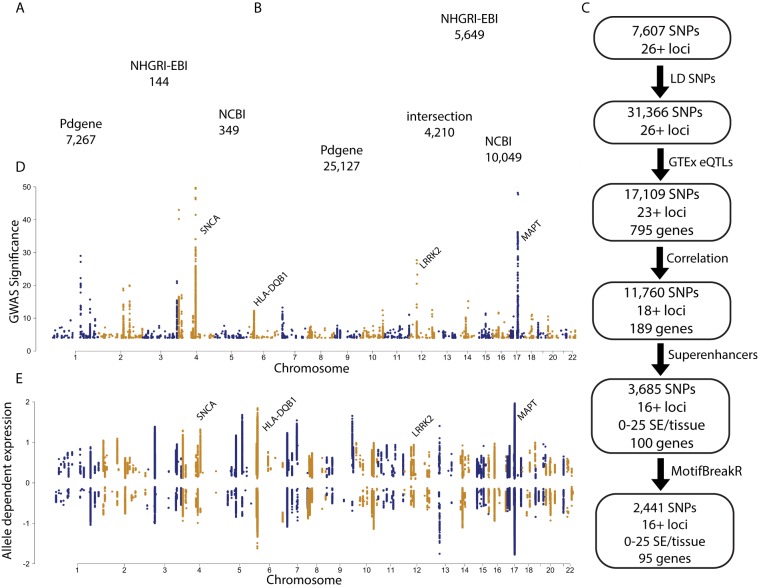
Study Design. (A) Euler diagram of 7,607 risk SNPs linked to PD obtained from three sources. Numbers represent the number of variants number in the entire set. (B) Surrogate variants in LD (r^2^ > 0.8) with risk SNPs were added to obtain 31,366 unique SNPs. (C) Flow chart of study design, (detail found in S1–S3 Files). (D) Manhattan plot of associated risk significance for 31,366 SNPS. (E) Manhattan plot of 17,109 SNPs which have an associated significant eQTL measurement in any of 53 tissues from GTEx.

We obtained 7,607 PD risk SNPs along with an additional 23,759 surrogate polymorphisms at r^2^ > 0.8 occupying at least 26 loci (Fig 1A–1C, Methods). It is obvious that the resulting 31,366 SNPs needed to be reduced to a more manageable number for hypotheses generation, as well as to associate risk with specific genes and regulatory elements.

We first reduced the set of potential PD risk SNPs by removing those which were not associated with known gene expression changes. The most direct way to link SNPs with gene expression is through variant-RNA expression association known as expression quantitative trait loci (eQTL) screens [7]. This is achieved by associating significant risk SNPs plus surrogates to genes by searching across GTEx-significant eQTLs and combining the results from each of 53 tissues; this generated a total of 795 genes (Fig 1D). Some risk loci queries (chromosomes 8, 18, 19) resulted in no eQTLs in the GTEx database, while other loci show association with a large number of genes. Large signals were seen at several regions, including chromosome 6 and 17. The largest number of eQTL genes (egenes) as well as the strongest change in expression were seen at chromosome *17q21*. This locus, as well as that at chromosome *6p21*, is located in a region with a great many polymorphisms in high LD.

We compared the set of 795 SNP/gene associations based on GTEx eQTLs with other associations based on the nearest transcription start site (TSS); on all TSS located within 1Mb at the gene locus given by the database (typically but not always the closest gene body). We used gene set enrichment to compare the sets produced by the different methods. In order to examine whether any functional pathways were over-represented in these gene sets, we used pathway enrichment and found that nearly all gene sets showed a similar functional enrichment of antigen-related processes (data not shown). Upon further examination, it became obvious that the *6p21*.*32* locus which contain a large linkage disequilibrium (LD) block that includes many of the *HLA*-MHC class I and class II protein coding genes—overwhelmed other enrichment signals. Although some non-*HLA*-related processes were enriched, in nearly every PD risk-SNP/gene set we examined, antigen presentation pathways predominated.

**Fig 2:**
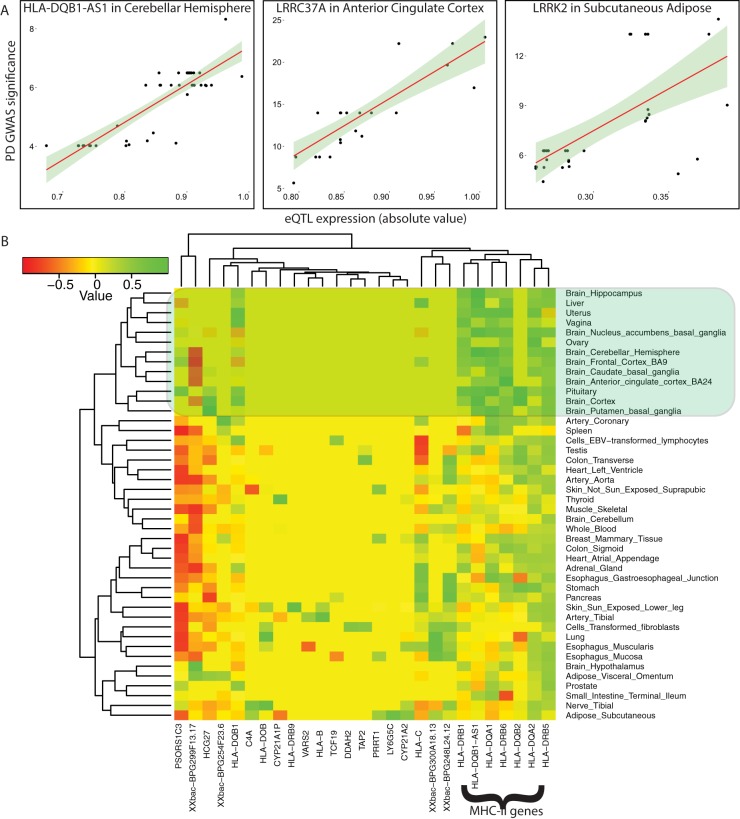
Correlation between GWAS significance and eQTL expression level. (A) Sample plots of the relationship between GWAS significance and eQTL expression for genes at the *HLA*, *LRRK2*, and *MAPT* loci. (B) Heatmap of positive correlation (green) coefficients for 27 genes at the *HLA* locus.

Although, neuorinflammation generally, and the *HLA* MHC genes specifically, have been related to Parkinson’s disease [8–11], it is still possible that the functional enrichment is spurious. The *HLA* locus is a highly polymorphic site having a large number of variants in high LD and containing genes with related function, any PD risk variants within will be indirectly associated with *HLA* genes due to linkage with surrogate polymorphisms. In order to test whether the measured eQTL associations, including those at *6p21*, directly impact PD risk we measured and found strong correlation between associated eQTL expression changes and PD GWAS significance of variants for individual genes (Fig 2). Filtering by these correlations reduced the PD risk associated genes to 189. Again, GSEA implicated MHC II genes, and to a lesser degree, MHC I genes.

Because most risk variants lie in noncoding DNA regions, we have reasonable confidence that variants affect phenotype by altering gene transcription, most likely by disrupting regulatory elements (REs). A particularly active subset of REs is situated in super-enhancers (SEs). SEs are large clusters of enhancer elements which show high association for Mediator occupancy and histone H3K27Ac modifications, and they show more tissue specific activity than enhancers in general [12–14]. As we and others have reported, we found that candidate risk SNPs often overlap SEs, and may especially affect disease predisposition. By further sub-classification of the risk SNPs according to overlap with dbSUPER defined superenhancers, we reduced the number of risk variants to 3685 and of risk genes to 100, with chromosome 6 and chromosome 17 having 1063 and 1322 variants, respectively.

Finally, we have previously shown that locating putative transcription factor (TF) binding motif disruption is an effective way to identify possible functional risk SNPs [15]. We filtered the SE SNP set for TF motif disruption and found that the associated gene sets changed little, falling to 95 genes (S3 File), and indicating that the previous subsetting by super-enhancer overlap had enriched for TF binding motifs. Correspondingly the significant GSEA signals related to the locus at chromosome *6p21*.*32*. The most significant gene ontology categories were by Panther, “MHC class II receptor activity” (p = 5.8e-4), MetaCore from Thomson Reuters: “peptide antigen binding” (p = 4.906e-112), and David, “interferon-gamma-mediated signaling pathway” (p = 1.97E-7) [16, 17].

## Discussion

Pdgene currently curates the most comprehensive GWAS of PD risk, having imputed association scores for 7.9 million variants [18]. To maximize the possible network of associated risk genes we used a low (0.001) p-value to obtain 7267 SNPs from pdgene.org and added to that risk SNPs from the NIH GWAS Catalog and NCBI PheGenI databases. Associating risk SNPs and LD SNPs to genes from the GTEx eQTL data gave a total of 795 genes (527 protein coding genes) across all 53 tissues. The median number of significant egenes per variant was 4 genes per tissue (max 18). Brain specific eQTLs consisted of 175 genes. This much smaller gene set may better predict influential genes, but non-brain tissues may have also a role in PD development and progression [6]. The smaller subset of brain-tissue egenes included 20 genes at the *HLA* locus (chromsome 6) and 28 genes at the *MAP*T locus (chromosome 17). This agrees with results from a similar study that examined 67 PD SNPs in a small number of PD and control brain cortical samples and found cis eQTLs at the same two regions [19].

GTEx gene expression data can be oriented by allele frequency and it is worth noting that some signals are directional in this presentation, indicating that the variants which show a positive expression changes are primarily the major or minor population alleles, and that LD variants all affect the same gene or genes. For instance, the signal on chromosome 12 for *LRRK2* [MIM 609007] is primarily in the positive direction, indicating all significant nearby alleles are the low-frequency, minor alleles (in this case the minor alleles are the risk alleles) and these correlate with higher *LRRK2* expression relative to the major allele. This is made more obvious by multiplying the GWAS–log(p. value) by eQTL expression (S1 Fig). The minor risk alleles are associated with increased *LRRK2*, which may indicate that a slightly elevated *LRRK2* level leads to a small but cumulative relative PD risk. This is consistent with findings that overexpression of wild-type or mutant *LRRK2* induces neural toxicity and elevated protein levels or activity that possibly contribute to Parkinson’s disease [20–22]. In other regions, the signal is more complex, and in these cases, a single allele may be associated with an increase in one gene transcript but a decrease in another.

Because eQTL studies produce a quantitative association between gene expression and multiple polymorphisms it may be possible to prioritize SNPs by comparing the expression level changes. A large change in gene expression may be more tractable experimentally and so can be used to prioritize targets. A corollary and proof of principle is that SNPs which alter the same genes by different amounts should show a corresponding difference in GWAS significance. Indeed, we see more positive correlation and less negative correlation than expected by chance in pairwise SNP comparisons of the difference in eQTL effect sizes and GWAS measured significance (S2 Fig). This principle is noisier (due to fewer values) but more direct by searching for genes that show a positive correlation between GWAS significance and eQTL expression. GWAS significance has been previously positively correlated with eQTL effect size [23]. We used this method to reduce the PD-associated genes from 795 to 189. However, for many genes the number of SNPs with relevant measurement is low and so correlation is inaccurate. Furthermore, we only consider significant p-values and significant eQTLs further increasing the accuracy of correlation. For these reasons, we chose a correlation cutoff of 0.4 to increase the sensitivity. We believe this set of genes includes those for which there is the greatest evidence for involvement with PD etiology, although some important genes, including *SNCA*, were removed.

We hypothesize that some GWAS signals are due to complex haplotypes containing multiple linked SNPs, which confer risk through synergistic disruption of multiple TF binding sites simultaneously. To maximize the identification of these cases and to identify specific regulatory elements we sub-classified the set of risk SNPs by overlap with super-enhancers, the large enhancer regions showing unusually high active histone signals. In this way, we reduced the number of risk gene candidates to 100. After further filtering SNPs for those that disrupt TF-binding protein motifs, the median number of egenes per SNP per tissue was 2 (max 14). This set of 95 genes is functionally enriched for antigen presentation processes.

In general, gene set enrichment for functionally related genes following our filtering of PD risk associations, pointed to fewer underlying processes than expected. For instance, the set of genes curated by the Parkinson's Disease Gene Ontology Annotation Initiative displays broader biological functions [24]. This disparity may reflect inadequate power or unsuccessful subgrouping in the underlying GWAS studies. The functional enrichment seen here is due to a single locus containing multiple *HLA* genes. However, one must be careful over-interpreting GSEA for locations where closely spaced genes share similar functions. GSEA typically assumes multiple independent random variables. However, this assumption is not valid when a single variant can affect multiple related genes such as at the *HLA* region, which has previously shown strong correlation in MHC gene expression [25]. Furthermore, the density of variants near the *HLA* genes is high, making it more likely that noise alone will provide signals in these regions. Never-the-less, the strong GSEA for MHC-II genes (and significant enrichment for MHC-I genes) does indicate that variants in these regions can strongly affect multiple genes in a single pathway. Furthermore, in a previous study that linked genes to risk SNPs by proximity rather than by eQTLs, an enrichment for immunological gene categories remained even when the HLA locus was treated as a single signal [26].

**Fig 3:**
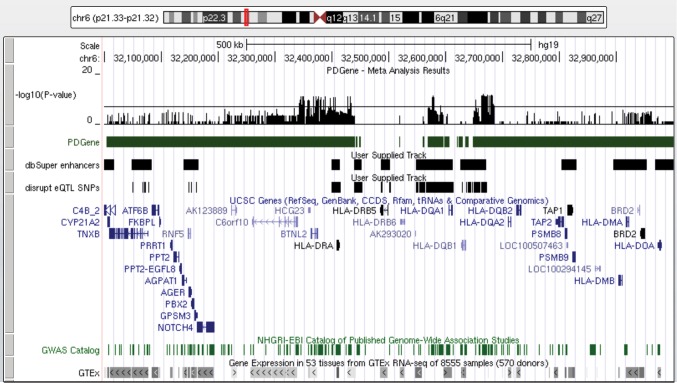
UCSC browser view of MHC-II *HLA* locus. GWAS p values are from Nalls et. al [3]. A total of 595 PD risk SNPs that overlap dbSUPER defined super-enhancers, show eQTLs, and disrupt a TF binding motif (labeled disrupt eQTL SNPs, S1 File) are shown.

The *6p21*.*32* risk locus contains approximately 3300 risk SNPs in LD (r^2^>0.8) and spans close to 2Mb and 121 genes. Cis eQTL analyses indicate that allele specific expression may be altered in up to 60 genes in this region, providing associated allele-specific differences in many traits and disorders. Indeed, the GWAS catalog links this locus with some 175 traits, approximately 10% of studied parameters [4]. Removing associations with genes that show lower PD correlation coefficients than overlapping variants associated with an eQTL in any tissue with dbSUPER super-enhancers, reduced the number of variants to 1063 with associated expression changes in 15 genes. We analyzed these with MotifbreakR [15] to find variants which alter putative TF-binding motifs; 595 risk polymorphisms strongly disrupted 79 Factorbook binding motifs (chromosome 6 summarized as a tract in Fig 3 and disrupted TFs listed in S1 File). We realize that this shorter list of variant/gene associations is by no means comprehensive, but it does represent the strongest indication of PD etiology.

A high GWAS significance may indicate one of three things. Firstly, the expression of a very central protein can be affected, perhaps only slightly. *SNCA* is a likely candidate for this type of risk. Secondly, a peripheral process may be affected, but to a very high degree. The large eQTL signals at the *HLA* and *MAPT* loci point to such relationships. Lastly, multiple peripheral processes may be affected simultaneously. This is particularly likely in large LD blocks where multiple correlated variants can have distinct effects. Again, both the *MAPT* and *HLA* may fall in this category.

We expect that multiple variants near the MHC-II genes and which overlap one of several super-enhancer regions (Fig 3), independently affect tissue-specific MHC-II gene expression levels and likely act to synergistically alter adaptive immunity-related processes. By comparing the associated eQTL signals across tissues we found that PD risk alleles at this locus are associated with increased expression of 7 HLA-genes (*HLA-B*, *HLA-C*, *HLA-DQA1*, *HLA-DQB1*, *HLA-DQB1-AS1*, *HLA-DRB1*, *HLA-DRB5*) and decreased expression of 4 genes (*HLA-DOB*, *HLA-DQA2*, *HLA-DQB2*, *HLA-DRB6*). MHC-II genes are expressed by antigen presenting cells, including microglia. The number of MHC-II-expressing cells increases with neuroinflammation and PD patients show more activated microglia then controls [27–29]. Activated microglia help clear debris, both foreign and native (including dying neurons), and produce pro-inflammatory factors. In addition, it was shown that microglial MHC-II plays a central role in the activation of both the innate and adaptive immune responses to alpha-synuclein expression, a hallmark in PD progression [30]. Altered antigen presentation pathways associated with risk alleles may contribute to prolonged neuro-inflammation or otherwise increase the loss of dopaminergic neurons in PD and as such be related to the microbiome in the gut [31]. The large number of variants in this region may relate to a highly complex regulation coupled with selective pressure on these genes and imply an importance of the MHC-II processes for many disorders including Parkinson’s disease. However, we cannot rule out a significant role for MHC-I pathways in PD. Interestingly the density of overall GWAS catalog risk SNPs aligns well with super-enhancer-located PD SNPs (Fig 3) suggesting that a relatively small number of SNPs may have diverse and complex effects on a variety of disorders and that examining a single index SNP is not a good model for causality.

## Supporting information

S1 Fig *LRRK2* eQTL associated variants. GWAS significance multiplied by 100 + eQTL beta and plotted by chromosome position for each variant.
(EPS)

S2 Fig Greater positive correlation exists between GWAS significance and eQTL in SNP-SNP pairwise comparisons then expected by chance. (A) Plot of correlation per tissue per gene for all SNP-SNP pairwise comparisons. (B) Q-Q plot for correlation scores for tissue-gene sets with greater than 100 SNP-SNP comparisons.
(EPS)

S1 File GWAS PD SNPs. 2,441 SNPs which disrupt putative binding motifs.
(TXT)

S2 File SNP locations. Chromosomal locations of SNPs which disrupt binding motifs.
(BED)

S3 File Integrated GWAS and eQTL PD data. 795 egenes associated with PD risk with annotations indicating each additional filtering step.
(TXT)

## References

[1] Driver JA, Logroscino G, Gaziano JM, Kurth T. Incidence and remaining lifetime risk of Parkinson disease in advanced age. Neurology. 2009;72(5):432–8. PubMed Central PMCID: PMCPMC2676726. doi: 10.1212/01.wnl.0000341769.50075.bb 19188574 10.1212/01.wnl.0000341769.50075.bb PMC2676726

[2] Labbe C, Lorenzo-Betancor O, Ross OA. Epigenetic regulation in Parkinson's disease. Acta Neuropathol. 2016;132(4):515–30. PubMed Central PMCID: PMCPMC5026906. doi: 10.1007/s00401-016-1590-9 27358065 10.1007/s00401-016-1590-9 PMC5026906

[3] Nalls MA, Pankratz N, Lill CM, Do CB, Hernandez DG, Saad M, et al Large-scale meta-analysis of genome-wide association data identifies six new risk loci for Parkinson's disease. Nat Genet. 2014;46(9):989–93. PubMed Central PMCID: PMCPMC4146673. doi: 10.1038/ng.3043 25064009 10.1038/ng.3043 PMC4146673

[4] Welter D, MacArthur J, Morales J, Burdett T, Hall P, Junkins H, et al The NHGRI GWAS Catalog, a curated resource of SNP-trait associations. Nucleic Acids Res. 2014;42(Database issue):D1001–6. PubMed Central PMCID: PMCPMC3965119. doi: 10.1093/nar/gkt1229 24316577 10.1093/nar/gkt1229 PMC3965119

[5] Soldner F, Stelzer Y, Shivalila CS, Abraham BJ, Latourelle JC, Barrasa MI, et al Parkinson-associated risk variant in distal enhancer of alpha-synuclein modulates target gene expression. Nature. 2016;533(7601):95–9. PubMed Central PMCID: PMCPMC5042324. doi: 10.1038/nature17939 27096366 10.1038/nature17939 PMC5042324

[6] Coetzee SG, Pierce S, Brundin P, Brundin L, Hazelett DJ, Coetzee GA. Enrichment of risk SNPs in regulatory regions implicate diverse tissues in Parkinson's disease etiology. Sci Rep. 2016;6:30509 PubMed Central PMCID: PMCPMC4962314. doi: 10.1038/srep30509 27461410 10.1038/srep30509 PMC4962314

[7] Consortium GT. The Genotype-Tissue Expression (GTEx) project. Nat Genet. 2013;45(6):580–5. PubMed Central PMCID: PMCPMC4010069. doi: 10.1038/ng.2653 23715323 10.1038/ng.2653 PMC4010069

[8] Hamza TH, Zabetian CP, Tenesa A, Laederach A, Montimurro J, Yearout D, et al Common genetic variation in the HLA region is associated with late-onset sporadic Parkinson's disease. Nat Genet. 2010;42(9):781–5. PubMed Central PMCID: PMCPMC2930111. doi: 10.1038/ng.642 20711177 10.1038/ng.642 PMC2930111

[9] International Parkinson Disease Genomics C, Nalls MA, Plagnol V, Hernandez DG, Sharma M, Sheerin UM, et al Imputation of sequence variants for identification of genetic risks for Parkinson's disease: a meta-analysis of genome-wide association studies. Lancet. 2011;377(9766):641–9. PubMed Central PMCID: PMCPMC3696507. doi: 10.1016/S0140-6736(10)62345-8 21292315 10.1016/S0140-6736(10)62345-8 PMC3696507

[10] Kannarkat GT, Cook DA, Lee JK, Chang J, Chung J, Sandy E, et al Common Genetic Variant Association with Altered HLA Expression, Synergy with Pyrethroid Exposure, and Risk for Parkinson's Disease: An Observational and Case-Control Study. NPJ Parkinsons Dis. 2015;1. PubMed Central PMCID: PMCPMC4853162. 10.1038/npjparkd.2015.2 PMC4853162 27148593

[11] Wang Q, Liu Y, Zhou J. Neuroinflammation in Parkinson's disease and its potential as therapeutic target. Transl Neurodegener. 2015;4:19 PubMed Central PMCID: PMCPMC4603346. doi: 10.1186/s40035-015-0042-0 26464797 10.1186/s40035-015-0042-0 PMC4603346

[12] Khan A, Zhang X. dbSUPER: a database of super-enhancers in mouse and human genome. Nucleic Acids Res. 2016;44(D1):D164–71. PubMed Central PMCID: PMCPMC4702767. doi: 10.1093/nar/gkv1002 26438538 10.1093/nar/gkv1002 PMC4702767

[13] Niederriter AR, Varshney A, Parker SC, Martin DM. Super Enhancers in Cancers, Complex Disease, and Developmental Disorders. Genes (Basel). 2015;6(4):1183–200. PubMed Central PMCID: PMCPMC4690034. 26569311 10.3390/genes6041183 PMC4690034

[14] Quang DX, Erdos MR, Parker SC, Collins FS. Motif signatures in stretch enhancers are enriched for disease-associated genetic variants. Epigenetics Chromatin. 2015;8:23 PubMed Central PMCID: PMCPMC4502539. doi: 10.1186/s13072-015-0015-7 26180553 10.1186/s13072-015-0015-7 PMC4502539

[15] Coetzee SG, Coetzee GA, Hazelett DJ. motifbreakR: an R/Bioconductor package for predicting variant effects at transcription factor binding sites. Bioinformatics. 2015;31(23):3847–9. PubMed Central PMCID: PMCPMC4653394. doi: 10.1093/bioinformatics/btv470 26272984 10.1093/bioinformatics/btv470 PMC4653394

[16] Huang da W, Sherman BT, Lempicki RA. Systematic and integrative analysis of large gene lists using DAVID bioinformatics resources. Nat Protoc. 2009;4(1):44–57. doi: 10.1038/nprot.2008.211 19131956 10.1038/nprot.2008.211

[17] Mi H, Poudel S, Muruganujan A, Casagrande JT, Thomas PD. PANTHER version 10: expanded protein families and functions, and analysis tools. Nucleic Acids Res. 2016;44(D1):D336–42. PubMed Central PMCID: PMCPMC4702852. doi: 10.1093/nar/gkv1194 26578592 10.1093/nar/gkv1194 PMC4702852

[18] Lill CM, Roehr JT, McQueen MB, Kavvoura FK, Bagade S, Schjeide BM, et al Comprehensive research synopsis and systematic meta-analyses in Parkinson's disease genetics: The PDGene database. PLoS Genet. 2012;8(3):e1002548 PubMed Central PMCID: PMCPMC3305333. doi: 10.1371/journal.pgen.1002548 22438815 10.1371/journal.pgen.1002548 PMC3305333

[19] Latourelle JC, Dumitriu A, Hadzi TC, Beach TG, Myers RH. Evaluation of Parkinson disease risk variants as expression-QTLs. PLoS One. 2012;7(10):e46199 PubMed Central PMCID: PMCPMC3465315. doi: 10.1371/journal.pone.0046199 23071545 10.1371/journal.pone.0046199 PMC3465315

[20] Li JQ, Tan L, Yu JT. The role of the LRRK2 gene in Parkinsonism. Mol Neurodegener. 2014;9:47 PubMed Central PMCID: PMCPMC4246469. doi: 10.1186/1750-1326-9-47 25391693 10.1186/1750-1326-9-47 PMC4246469

[21] Skibinski G, Nakamura K, Cookson MR, Finkbeiner S. Mutant LRRK2 toxicity in neurons depends on LRRK2 levels and synuclein but not kinase activity or inclusion bodies. J Neurosci. 2014;34(2):418–33. PubMed Central PMCID: PMCPMC3870929. doi: 10.1523/JNEUROSCI.2712-13.2014 24403142 10.1523/JNEUROSCI.2712-13.2014 PMC3870929

[22] Tsika E, Moore DJ. Mechanisms of LRRK2-mediated neurodegeneration. Curr Neurol Neurosci Rep. 2012;12(3):251–60. doi: 10.1007/s11910-012-0265-8 22441981 10.1007/s11910-012-0265-8

[23] Zhu Z, Zhang F, Hu H, Bakshi A, Robinson MR, Powell JE, et al Integration of summary data from GWAS and eQTL studies predicts complex trait gene targets. Nat Genet. 2016;48(5):481–7. doi: 10.1038/ng.3538 27019110 10.1038/ng.3538

[24] Foulger RE, Denny P, Hardy J, Martin MJ, Sawford T, Lovering RC. Using the Gene Ontology to Annotate Key Players in Parkinson's Disease. Neuroinformatics. 2016;14(3):297–304. PubMed Central PMCID: PMCPMC4896971. doi: 10.1007/s12021-015-9293-2 26825309 10.1007/s12021-015-9293-2 PMC4896971

[25] Michalopoulos I, Pavlopoulos GA, Malatras A, Karelas A, Kostadima MA, Schneider R, et al Human gene correlation analysis (HGCA): a tool for the identification of transcriptionally co-expressed genes. BMC Res Notes. 2012;5:265 PubMed Central PMCID: PMCPMC3441226. doi: 10.1186/1756-0500-5-265 22672625 10.1186/1756-0500-5-265 PMC3441226

[26] Holmans P, Moskvina V, Jones L, Sharma M, International Parkinson's Disease Genomics C, Vedernikov A, et al A pathway-based analysis provides additional support for an immune-related genetic susceptibility to Parkinson's disease. Hum Mol Genet. 2013;22(5):1039–49. PubMed Central PMCID: PMCPMC3561909. doi: 10.1093/hmg/dds492 23223016 10.1093/hmg/dds492 PMC3561909

[27] Zhao Y, Forst CV, Sayegh CE, Wang IM, Yang X, Zhang B. Molecular and genetic inflammation networks in major human diseases. Mol Biosyst. 2016;12(8):2318–41. PubMed Central PMCID: PMCPMC4955784. doi: 10.1039/c6mb00240d 27303926 10.1039/c6mb00240d PMC4955784

[28] Rocha NP, de Miranda AS, Teixeira AL. Insights into Neuroinflammation in Parkinson's Disease: From Biomarkers to Anti-Inflammatory Based Therapies. Biomed Res Int. 2015;2015:628192 PubMed Central PMCID: PMCPMC4532803. doi: 10.1155/2015/628192 26295044 10.1155/2015/628192 PMC4532803

[29] Hirsch EC, Vyas S, Hunot S. Neuroinflammation in Parkinson's disease. Parkinsonism Relat Disord. 2012;18 Suppl 1:S210–2. 22166438 10.1016/S1353-8020(11)70065-7

[30] Harms AS, Cao S, Rowse AL, Thome AD, Li X, Mangieri LR, et al MHCII is required for alpha-synuclein-induced activation of microglia, CD4 T cell proliferation, and dopaminergic neurodegeneration. J Neurosci. 2013;33(23):9592–600. PubMed Central PMCID: PMCPMC3903980. doi: 10.1523/JNEUROSCI.5610-12.2013 23739956 10.1523/JNEUROSCI.5610-12.2013 PMC3903980

[31] Sharon G, Sampson TR, Geschwind DH, Mazmanian SK. The Central Nervous System and the Gut Microbiome. Cell. 2016;167(4):915–32. PubMed Central PMCID: PMCPMC5127403. doi: 10.1016/j.cell.2016.10.027 27814521 10.1016/j.cell.2016.10.027 PMC5127403

