# Correction: Parkinson's disease-associated genetic variation is linked to quantitative expression of inflammatory genes

**DOI:** 10.1371/journal.pone.0210931

**Published:** 2019-01-14

**Authors:** The PLOS ONE Staff

There are errors in Fig 1. Venn diagram circles are missing from Fig 1A and 1B. Please see the complete, corrected version of Fig 1 here. The publisher apologizes for this error.

**Fig 1:**
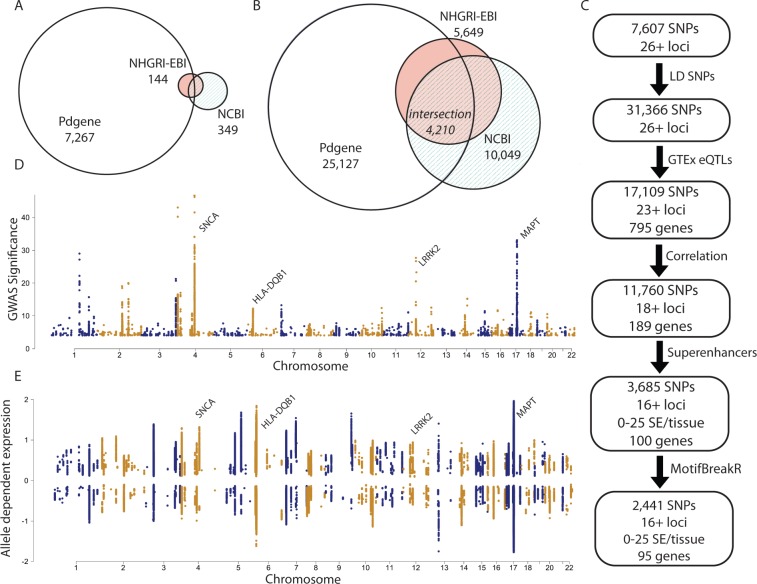
Study design. (A) Euler diagram of 7,607 risk SNPs linked to PD obtained from three sources. Numbers represent the number of variants number in the entire set. (B) Surrogate variants in LD (r^2^ > 0.8) with risk SNPs were added to obtain 31,366 unique SNPs. (C) Flow chart of study design, (detail found in S1–S3 Files). (D) Manhattan plot of associated risk significance for 31,366 SNPS. (E) Manhattan plot of 17,109 SNPs which have an associated significant eQTL measurement in any of 53 tissues from GTEx.
